# Supplementary material for: Managing Irinotecan-Induced Diarrhea: A Comprehensive Review of Therapeutic Interventions in Cancer Treatment
Source: Pharmaceuticals (Basel). 2025 Mar 2;18(3):359. doi: 10.3390/ph18030359 (PMC11944746; doi:10.3390/ph18030359)
Supplement: Supplementary file 1 [file pharmaceuticals-18-00359-s001.zip › pharmaceuticals-3465879-supplementary.pdf]

Supporting information

Managing Irinotecan-Induced Diarrhea: A Comprehensive Review of Therapeutic Interventions in Cancer Treatment

Xiaoqin Yang[a], Jiamei Chen[a],Yitao Wang[b], Yihan Wu[a]* and Jinming Zhang[a]*

a State Key Laboratory of Southwestern Chinese Medicine Resources, School of Pharmacy, Chengdu University of Traditional Chinese Medicine, Chengdu 611137, China

b State Key Laboratory of Quality Research in Traditional Chinese Medicine, Institute of Chinese Medical Sciences, University of Macau, Macau 999078, China.

**Corresponding author:*

Dr. Yihan Wu, No.1166 Liutai Avenue,Wenjiang District, Chengdu city, State Key Laboratory of Southwestern Chinese Medicine Resources, College of Pharmacy, Chengdu University of Traditional Chinese Medicine, Chengdu, China

Tel.: +86 15680803903

E-mail address: yihanwuone@126.com

Dr. Jinming Zhang, No.1166 Liutai Avenue,Wenjiang District, Chengdu city, State Key Laboratory of Southwestern Chinese Medicine Resources, College of Pharmacy, Chengdu University of Traditional Chinese Medicine, Chengdu, China

Tel.: +86 13551043885

E-mail address:cdutcmzjm@126.com

Table S1 Major chemotherapeutic agents for the treatment of CPT-11-induced diarrhea (Guide recommendation)

Section NO.	Chemotherapeutic agents	Possible mechanisms of action	Limitations and Comments	References
1.1.1.1	Loperamide	Acts at μ-opioid receptor; slows intestinal peristalsis; anti-secretory effects through inhibition of TXA2	Aggressive treatment with loperamide from diarrhea symptoms is the most effective strategy. But once CID fulminant rarely succeeful.	[59,60]
1.1.1.2	Octreotide	Reduced the secretion of specific gut hormones; prolongates intestinal transit time; promotes intestinal absorption of fluids and electrolytes	Recommended second-line treatment strategy. Reduced intestinal transport can lead to more intestinal damage. Lack of high-quality research on prevention.	[61-63]
1.1.1.3	Acetorphan	Enkephalinase inhibitor, antidiarrheal activity and antisecretory mechanism	Failed to show activity as prophylactic agent.	[64,65]
1.1.1.4	Budesonide	Anti-inflammatory; Topically active corticosteroid that might restore mucosal function and fluid absorption; a 90% first-pass effect in the liver results in low systemic availability	Can be recommended. However, the safety data in the clinical environment are not clear. Can cause side effects such as dry mouth, stomach discomfort, bad taste ect.	[66,67]
1.1.2	Bicarbonate powder	Alkalize intestinal, changing the equilibrium of carboxylate isomers and reduce the toxicity of CPT-11	Daily consumption of highly alkalized 2～3 L water per day is cumbersome. Effective with defecation control but patient compliance and rationality will be a problem.	[70,71]
1.1.3.1	Cyclosporine	inhibited ABCB1, ABCC2 and ABCG2 to decrease in CPT-11 biliary excretion	Cyclosporine toxicity.<br>Prophylaxis not complete.	[50,75]
1.1.3.2	Probenecid	ABCC2 inhibition	No tested in clinical environment. Can itself cause gastrointestinal toxicity.	[73,74]
1.1.4.1	SAL	β-glucuronidase inhibitor	Not fully effective clinically and no testing in clinical environment.	[79]
1.1.4.2	Chrysin	UGT1A1 upregulator	CID still 10%. Results of phase II awaited.	[82,83]

1.1.4.3	hiCE inhibitors	Inhibited Intestinal carboxylesterase	Results awaited in preclinical models. Still a long time to know clinical benefit.	[86-88]
1.1.4.4	Anti-epileptic drugs	CYP3A4 inducers	CPT-11's MTD has tripled. However, the therapeutic index of antiepileptic drugs is narrow. Therefore, caution should be taken	[89]
1.1.4.5	Celecoxib	Inhibited COX-2and PGE2	Encouraging activity shown in preclinical models absent in clinical trials.	[91-93]
1.1.5.1	Probiotics	Inhibit β-glucuronidase activity, epithelial cell proliferation, reduce intestinal cell apoptosis, prevent the increase of goblet cell number and mucin secretion.	Although only moderately effect, but cheap, non-toxic and convenient.	[94-95]
1.1.5.2	Antibiotics	reduced activity of bacterial *β*-glucuronidase. Streptomycin can also reduce the intestinal absorption of CPT-11 and CES, and increase the UGT activity of intestinal epithelium.	Feasible but the additional antibiotic toxicity is a problem. Including fungal infections caused by the removal of symbiotic bacteria and toxicity caused by antibiotics.	[97-102]
1.1.6.1	Activated charcoal	Decreased CPT-11 absorption	Incomplete effect, can absorb other orally administered comedications and is cumbersome due to delivery three times per day.	[103,104]
1.1.6.2	AST-120	Decreased CPT-11 absorption	Incomplete effect, can absorb other orally administered comedications and is cumbersome due to delivery three times per day.	[105,106]
1.1.7.1	Thalidomide	Inhibited TNF-α, IL-1β, IL-6 and IFN-γ, inhibition of ABCB1, ABCC2.	Encouraging results have been shown in the prevention of CID, but further research is needed in this area.	[107-110]

1.1.7.2	Velafermin	Inhibited the production of intestinal inflammatory cytokines, reduce the apoptosis of intestinal epithelial cells, and regulate immunity and angiogenesis	Some doses seem to increase diarrhoea and mortality. In addition, FGF also plays a certain role in tumor growth. No tested in clinical trials.	[111]
1.1.7.3	IL-15	Inhibited apoptosis	No tested in clinical trials. In addition, the increase in MTD did not lead to an enhanced response in preclinical studies.	[112]
1.1.7.4	JBT-3002	Active IL-1α, IL-1β, IL-6, IL-15, TNF-α and nitric oxide	Not tested in clinical trials.	[113]
1.1.8.1	Oil supplementation	Mucosal protection	No tested in clinical trials. In the preclinical model improved the efficacy of CPT-11, but can not prevent intestinal toxicity.	[114]
1.1.8.2	L-Glutamine	Block the upregulation of β-glucuronidase and increase the ratio of reduced glutathione to oxidized glutathione.	Cumbersome as glutamine needs to be taken three or four times a day. No benefit in phase II trials.	[115]
1.1.8.3	phloroglucinol	Restore normal electrolytes, Selectively directly relax the smooth muscle cells of some organs	Incomplete effect.	[116,117]

Table S2 .Summary of the proved effects of Single TCM and active ingredients in CPT-11-induced diarrhea.

Name of TCM	Botanical latin name	Effective ingredients	Mechanisms of action	References
St. John's wort	*Hypericum perforatum*	Unkown	Alter CPT-11 and SN-38 pharmacokinetics; inhibit pro-inflammatory cytokines and intestinal epithelial apoptosis	[118,119]
Huanglian	*Coptis chinensis* Franch.	Berberine	Inhibit *β*-glucuronidase activity of intestinal bacteria	[120,121]
Jianghuang	*Curcuma Longa* L.	Curcumin	Inhibit NF- κB activation, oxidative stress and endoplasmic reticulum stress	[122,123]
Chenpi	*Citrμs reticμlata Blanco*	Hesperidin	Increase AUC5-t, decrease the clearance rate of CPT-11 and SN-38, reduce the excretion of CPT-11 and SN-38 in bile, selectively inhibit intestinal carboxylesterase	[124,125]

Table S3 .Summary of the proved effects of in TCM compound prescription CPT-11-induced diarrhea.

Name of formula	Botanical latin name	Ratio	Mechanisms of action	References
Huangqin decoction	*Scutellaria baicalensis* Georgi, *Glycyrrhiza uralensis* Fisch, *Paeonia lactiflora* Pall, *Ziziphus jujuba* Mill	3:2:2:2	Improve the structure of dysfunctional intestinal flora; up-regulate the expression of mucin and compact protein in intestinal epithelium; increase the content of NO in intestinal tissue and reduce the expression of PCNA; improve the changes of hydrophobic bile acid BAS to reduce the gastrointestinal toxicity induced by CPT11.	[126-128]
PHY906	*Scutellaria baicalensis* Georgi, *Glycyrrhiza uralensis* Fisch, *Paeonia lactiflora* Pall, *Ziziphus jujuba* Mill	Unkown	Promote the regeneration of intestinal cells or stem cells and several Wnt signaling components; reduce intestinal inflammation	[129-132]
Shengjiang Xiexin decoction	*Pinellia ternate*, *Glycyrrhiza uralensis*, *Coptis chinensis*, *Ziziphus jujuba, Zingiber officinale, Scutellaria baicalensis, Codonopsis pilosula, Zingiberis recens*	9:9:3:12:3:9:9:12	Inhibit intestinal cell apoptosis, promote intestinal cell proliferation, inhibit the activity of *β*-glucuronidase, improve the degree of intestinal mucosal injury, inhibit inflammatory reaction, and alleviate neutropenia.	[133-136]

Banxia Xiexin decoction	*Pinellia ternata* Breit., *Scutellaria baicalensis* Georgi, *Rhizoma Zingiberis*, *Salvia miltiorrhiza* Bunge, *Glycyrrhiza uralensis* Fisch, *Coptis chinensis* Franch , *Ziziphus jujuba* Mill	12:9:9:9:9:3:16	inhibit the expression of COX-2 in colon tissue and reduce the concentration of SN-38 in colon tissue	[137-139]
Gegen Qinlian decoction	*Pueraria lacei* Craib, *Scutellaria bai-calensis* Georgi, *Coptis chinensis* Franch, and *Glycyrrhiza uralensis* Fisch.	8:3:3:2	Inhibit carboxylipase activity; regulation of inflammatory factors and inhibition of the expression of antioxidant stress related proteins; through anti-inflammatory pathway, inhibition of neutrophil migration, antioxidation through Nrf2/Keap-1 pathway and enhancement of intestinal barrier function of colon	[140-142]
Xiao Chaihu deciction	*Bupleurum chinense*DC., *Scutellaria baicalensis* Georgi, *Panax ginseng* C. A. Mey., *Pinellia ternata,Glycyrrhiza uralensis* Fisch, *Zingiber officinale, Ziziphus jujuba* Mill.	8:3:3:3:3:3:4	Reduce the rate of blood stool and improve the injury of intestinal mucosa	[143-144]
Sishen Pill	*Psoralea corylifoliaL., Euodia rutaecarpa,Myristica fragrans* Houtt*.,Schisandra chinensis*	4:1:2:2:2	Decrease the activity of intestinal β-glucuronidase, reduce the level of IL-1β and TNF-α, and reduce the injury of intestinal mucosa.	[145]
Jiawei Xianglian decoction	*Coptis chinensis* Franch., *Aucklandia lappa,Sargentodoxa cuneate* Rehder ,*Taraxacum mongolicum Hand.*-Mazz , *Portulaca oleracea* L., *Euphorbia humifusa* Willd	2:3:5:4:10:5	Decrease the activity of *β*-glucuronidase，decrease the levels of TNF-*α*, IL-6, IL-1*β* and IL-15 inflammatory factors, and improve intestinal flora.	[146]
Senfu Zhulin San	*Panax ginseng* C. A. Mey., *Poria cocos, Atractylodes macrocephala* Koidz*.,Dioscorea opposita* Thunb., *Dolichos lablab* L., *Nelumbo nucifera* Gaertn., *Coix lacryma-jobi* L.var.ma-yuen, *Amomum villosum* Lour., *Platycodon grandiflorum,Glycyrrhiza uralensis* Fisch.	4:4:4:4:3:2:2:2:2:4	Regulate immune function and inhibit inflammatory response	[147]

Renshen Jianpi pill	*Panax ginseng* C. A. Mey., *Atractylodes macrocephala* Koidz.,*Poria cocos,Citrµs reticµlata Blanco*, *Aucklandia lappa* Decne., *Amomum villosum* Lour., *Astragalus membranaceus, Angelica sinensis,Ziziphus jujuba* Mill., *Polygala tenuifolia* Willd.	2:12:4:8:4:1:2:8:4:4:2	Balance the homeostasis of intestinal flora and reduce the levels of serum inflammatory factors of TNF-α, IL-6 and IL-1β	[148]
Hange-shashin-to	*Pinelliae tuber, Scutellariae radix, Glycyrrhizae radix, Zizyphi fructus, Ginseng radix, Coptidis rhizoma, Zingiberis Siccatum rhizoma*	10:5:5:5:5:2:5	Inhibit β-glucuronidase; reduce the production of prostaglandin E2 in colon; increase water absorption	[149]
Sairei-to	*Bupleuri radix, Pinelliae tuber, Alismatis rhizome,Scutellariae radix, Ginseng radix, Zizyphi fructus, Poria,Polyporus, Atractylodis lanceae rhizome, Cinnamomi cortex, Glycyrrhizae radix, Zingiberis rhizome*	7:5:5:3:3:3:3:3:3:2:2:1	Inhibit β-glucuronidase	[150,151]

Table S4 New irinotecan formulations with effects of modifications and references.

New Formulation	Effect of Modification	Reference
PEGylated liposomal irinotecan	Compared to administering irinotecan alone, the modified formulation of irinotecan demonstrated enhanced cytotoxicity in a murine model of brain metastasis	[155,156]
Hyaluronic Acid ChemoTransport (HyACT®)	The treatment has led to an increased sensitivity in tumor cells that are positive for CD44. Furthermore, when this approach is combined with standard irinotecan therapy, it has been shown to enhance progression-free survival rates in patients with metastatic colorectal cancer	[157-159]
Irinotecan (Iri)-fatty acid prodrugs (Iri5C, Iri-8C, and Iri-12C) with alkyl chains of different lengths synthesized by esterification using DCC (dicyclohexylcarbodiimide) and DMAP (4-dimethylamino-pyridine	The drug exhibited increased accumulation within cells, which corresponded to a heightened level of cytotoxicity for irinotecan	[160-161]
Cisplatin and irinotecan encapsulated in poly(d,l-lactide-co-glycolide)-co-poly(ethylene glycol) (PLGA–PEG)-based nanoparticles directed toward prostate cancer cellsoverexpressing PSMA receptors, by using PSMA ligand-S,S-2-(3-[5-amino-1-carboxypentyl]- ureido)pentanedioic acid	The targeted nanoparticles facilitate a selective internalization process through endocytosis and enable a regulated drug release mechanism. This approach allows the therapeutic compounds to function effectively as cytotoxic agents. The combined action of the two agents has demonstrated a synergistic effect, leading to an increased rate of cell elimination	[162]
Self-assemble poly(l-lysine)-b-poly(l-leucine) (PLys-b-PLeu) polymersomes.	No cytotoxic effects were observed in the examined cell lines, including HEK, NIH3T3, and A549, when exposed to the carriers. Furthermore, encapsulating irinotecan within polymersomes yielded comparable in vitro antitumor efficacy to the free drug form	[163]

Nanoparticle system prepared with poly(dl-lactic acid) (PLA), poly(ethylene glycol)-block-poly(propylene glycol)-block-poly(ethylene glycol) (PEG–PPG–PEG), and irinotecan.	The nanoparticles have shown a significant boost in their ability to fight Sarcoma 180, a solid tumor. They also possess the potential to be toxic to cancer cells in solid tumors that are not in the immediate vicinity of where they are administered	[164]

Table S5 New SN-38 formulations with effects of modifications and references.

New Formulation	Effect of Modification	Reference
PEGylated polyamidoamine (PAMAM) dendrimers containing SN-38 conjugated with peptides-BR2 and CyLoP1.	The developed formulation has proven to be significantly more cytotoxic against the murine colon carcinoma cell line CT26 than SN-38 in its unmodified state. In vivo studies have demonstrated that the formulation leads to an increased cellular uptake and heightened cytotoxic effect compared to administering SN-38 without modification	[144]
A liposome-based preparation of SN-38 (LE-SN-38)	LE-SN-38 has shown superior efficacy in enhancing cytotoxicity against a variety of tumor cell lines and in treating xenograft mouse models	[167]
Amorphous Solid Disodium Glycyrrhizinate and SN-38 Self-Assembled Micelles(Na2GA/SN-38-BM)	with favorable pharmacokinetics and distribution properties, demonstrating enhanced cytotoxicity against tumor cells and significant inhibition of tumor growth	[168]
SN-38 loaded on graphene oxides (GOs) modified with either polyvinylpyrrolidone (PVP) or excipient β-cyclodextrin (β-CD).	The nanocarrier-mediated delivery of SN-38 demonstrated an enhanced cytotoxic effect specifically in the MCF-7 breast cancer cell line	[169]

SN-38 conjugated to gold nanoparticles via oligonucleotides complementary to specific mRNAs unique to cancer cells of Ewing sarcoma.	The pharmaceutical compound was effectively targeted and precisely deployed in experimental settings encompassing both cellular (in vitro) and living organism (in vivo) conditions	[170]
PEGylated acetylated carboxymethylcellulose conjugate of SN-38 covalently attached it to an aptamer against CD133.	In vitro studies have shown that the HT29 cell line, known for its expression of CD133, exhibits a heightened absorption of the drug when it is delivered via a carrier. Additionally, the application of nanoconjugates has been found to significantly boost the drug's cytotoxicity, surpassing that of non-targeted, self-assembled nanoconjugates	[171]
